# A fragment based method for modeling of protein segments into cryo-EM density maps

**DOI:** 10.1186/s12859-017-1904-5

**Published:** 2017-11-13

**Authors:** Jochen Ismer, Alexander S. Rose, Johanna K. S. Tiemann, Peter W. Hildebrand

**Affiliations:** 1 Institute of Medical Physics and Biophysics, University Medicine Berlin, Charitéplatz 1, 10117 Berlin, Germany; 2 RCSB Protein Data Bank, San Diego Supercomputer Center, University of California, San Diego, CA 92093-0743 USA; 3 Institute of Medical Physics and Biophysics, University Leipzig, Härtelstraße 16-18, 04107 Leipzig, Germany

**Keywords:** Cryo-EM, Fragment based modeling, Flexible fitting

## Abstract

**Background:**

Single-particle analysis of electron cryo-microscopy (cryo-EM) is a key technology for elucidation of macromolecular structures. Recent technical advances in hardware and software developments significantly enhanced the resolution of cryo-EM density maps and broadened the applicability and the circle of users. To facilitate modeling of macromolecules into cryo-EM density maps, fast and easy to use methods for modeling are now demanded.

**Results:**

Here we investigated and benchmarked the suitability of a classical and well established fragment-based approach for modeling of segments into cryo-EM density maps (termed FragFit). FragFit uses a hierarchical strategy to select fragments from a pre-calculated set of billions of fragments derived from structures deposited in the Protein Data Bank, based on sequence similarly, fit of stem atoms and fit to a cryo-EM density map. The user only has to specify the sequence of the segment and the number of the N- and C-terminal stem-residues in the protein. Using a representative data set of protein structures, we show that protein segments can be accurately modeled into cryo-EM density maps of different resolution by FragFit. Prediction quality depends on segment length, the type of secondary structure of the segment and local quality of the map.

**Conclusion:**

Fast and automated calculation of FragFit renders it applicable for implementation of interactive web-applications e.g. to model missing segments, flexible protein parts or hinge-regions into cryo-EM density maps.

**Electronic supplementary material:**

The online version of this article (10.1186/s12859-017-1904-5) contains supplementary material, which is available to authorized users.

## Background

Cryo electron microscopy (cryo-EM) is a key technology for structural elucidation of molecular complexes. The vast majority of published cryo-EM density maps is resolved at medium resolutions between 6 and 9 Å or lower [1–3]. In these medium resolution maps, no side-chains are resolved, but secondary structure elements or backbone traces can be identified and modeled [4–6]. Recent technical advances in development of direct electron detectors significantly improved the resolution of structures determined by cryo-EM [7, 8]. Near atomic resolution of cryo-EM density maps now even allows de novo modeling of well-resolved parts [9]. However, flexible regions such as loops often remain unresolved [10]. In cases where conformational changes of proteins only affect a substructure of the protein or a single domain while the general fold remains unchanged, modeling focuses on the flexible hinge regions [11]. Approaches, where defined structural elements are modeled into an existing structural context are thus a regular part of the workflow to calculate structural coordinates from cryo-EM density-maps [10, 11]. Because of the wide range of structural biologists working in the field of cryo-EM, methods for modeling into cryo-EM density maps e.g. to be integrated by easy to use web services such as SL2 [12] can greatly enhance researcher productivity. Here we evaluate the applicability of a well established fragment based modeling approach [12–14] for prediction of protein segments into cryo-EM density maps. This novel method, termed FragFit, can be readily integrated into modeling approaches where e.g.: (i) conformational changes of proteins only affect a substructure of the protein or a single domain, while the general fold remains unchanged [11], (ii) parts in a protein model are missing [10], or (iii) where local flexibility does not allow unambiguous assignment of a single conformational state [15].

Several methods have been established for structure prediction of protein segments, especially for the purpose of loop modeling [13, 16, 17]. These methods can be divided into forcefield- [17] and fragment-based approaches [13]. Forcefield-based methods have the general advantage that, in principle, new polypeptide folds can be predicted. These tools are, however, computationally expensive [18], and are thus usually not applicable for instant visual control of the results in interactive web-applications. Fragment based methods allow for comparably fast assessment of results because searches leverage databases of pre-calculated fragments. The latter databases are typically either derived from third party databases of protein structures such as the Protein Data Bank (PDB) [12, 19] or from concatenating small fragments in a structural database [20, 21].

The quality of classical fragment based modeling depends on the algorithm used for fragment selection and on the completeness of the fragment database [22]. Since the number of conformations rises exponentially with the length of the segment, quality of prediction generally drops with segment length [23, 24]. Loops are structurally highly heterogeneous and flexible. Nevertheless, it has been suggested that the conformational space for loops up to 12–14 residues is covered by structural fragments derived from entries of the PDB [25, 26]. We therefore used LIP a regularly updated fragment database derived from the PDB for modeling of segments into cryo-EM density maps [12]. The advantage of this approach is that the segments derived from the PDB are taken from structures that have already been subject of a strict and independent quality control. To evaluate FragFit under realistic conditions we used experimentally derived cryo-EM density maps, which naturally include fragmentations and local variations in resolution, and excluded identical template fragments (with 90% sequence identity or higher to the queried segment) from modeling. We find FragFit to be a useful tool for quick and reliable modeling of segments of up to 20–25 residues length into cryo-EM density maps. Prediction quality depends on segment length, secondary structure type of the predicted segment and the local quality of the map.

## Methods

**Fig. 1:**
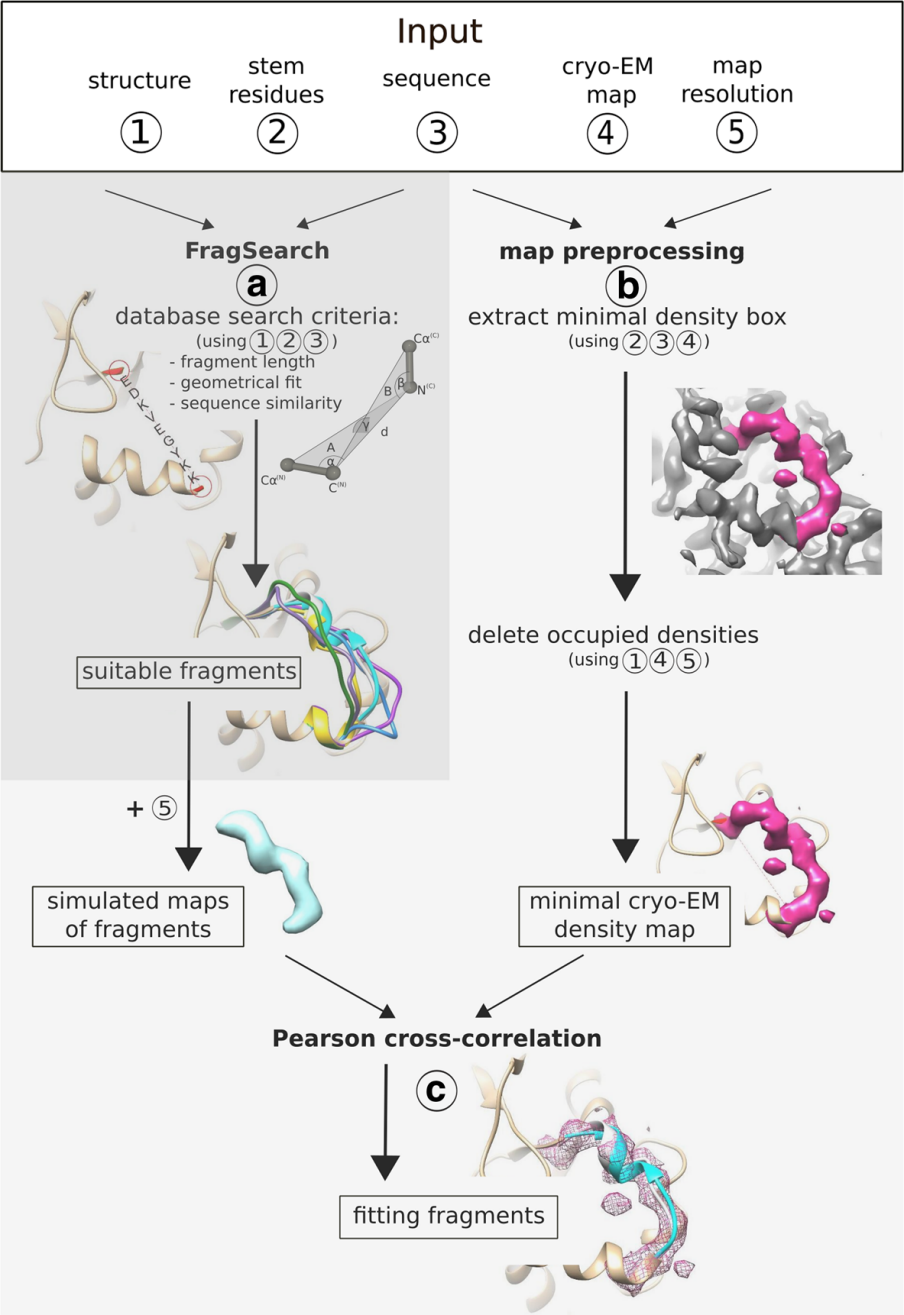
Workflow of FragFit. As input (top), (1) a PDB structure, (2) the stem atoms of residues flanking the queried segment, (3) the amino acid sequence of the queried segment and (4) the cryo-EM density map with (5) its resolution must be provided. **a** Sequence similarity between fragment and queried segment and matching of geometric fingerprints (Additional file 1: Figure S2) are used as evaluation criteria for FragSearch. **b** Cryo-EM density maps are preprocessed to minimize calculation time and to reduce false positive predictions. For that purpose, a minimal box limited to the maximum density of the missing segment is extracted and occupied densities are deleted. **c** Suitable fragments identified by FragSearch are re-scored by the Pearson cross-correlation coefficient between simulated and experimentally determined cryo-EM density maps, which selects for the best fitting fragments. All steps are presented in more detail in Additional file 1: Figure S6

To start a search, the amino acid sequence of the queried segment, the stem residues flanking the queried segment, the cryo-EM density map and its resolution must be provided (Fig. 1). The sequence similarity and a geometrical measure (termed geometric fingerprint) is used to search for suitable fragments (‘FragSearch’) in the fragment database derived from the RCSB PDB. These fragments are subsequently re-scored by their fit to preprocessed cryo-EM density maps to select for the best fitting fragments (‘FragFit’). Besides providing input arguments FragSearch and FragFit are fully automated procedures that do not require any intervention by the user.

### Fragment database and geometrical fingerprint

The fragment database LIP (‘Loops in Proteins’), which we employed to search for suitable fragments in the first prediction step (see Fig. 1a, ‘FragSearch’) contained about 9*10^8^ protein fragments. The database was composed of all overlapping fragments of 3–35 residues length extracted from about 100.000 entries of the PDB in June 2013. The number of fragments decreases linearly with fragment length, from about 23 to 19 million for fragments with 3 to 35 residues, respectively (see Additional file 1: Figure S1). With a recent update (February 2017) the database contains now more than 10^9^ protein fragments, extracted from more than 126.000 entries of the PDB. For each fragment the amino acid sequence, PDB identifier, chain identifier and the residue numbers of N- and C-terminal stem atoms is stored. In addition, a geometrical fingerprint is calculated for the stem atoms of each fragment (and also of the gap in the structure), composed of the distance d between the N- and C-terminal stem atoms and three angles defining their relative orientation (Fig. 1a, see Additional file 1: Figure S2). Matching of geometrical fingerprints of fragment and gap and sequence similarity (for details see [13]) are used as evaluation criteria by FragSearch (Fig. 1a).

### FragSearch

For detection of suitable fragments (FragSearch), we integrated the search algorithm of ‘SL2’ which is based on a hierarchical approach that minimizes calculation time (see [12–14]). First, fragments with the same number of amino acids as the missing segment and with a similar distance d of stem residues as in the gap (Δ*d* < 0.75 Å) are selected (see Additional file 1: Figure S2). Second, these fragments are ranked by the RMSD-value of their N- and C-terminal stem residues after superposition with the respective stem residues of the gap. Third, fragments whose incorporation would lead to clashes with other atoms of the same protein chain are identified and subsequently excluded. Moreover, fragments with identical primary structure or identical folds (with backbone RMSD <0.5 Å) are deleted (see [13]) to maximize the conformational space. In a fourth step, the top-1000 list of suitable candidates is re-ranked by sequence similarity to the queried segment and matching of geometrical fingerprints of fragment and gap (Fig. 1a). The top-100 list of suitable candidates is subsequently evaluated by FragFit, which employs cryo-EM density maps as an additional selection criterion.

### FragFit

The geometrical fit of the shape of a fragment to a cryo-EM density map is used to re-rank the top-100 list of suitable fragments and select for ‘fitting fragments’ (Fig. 1c). This fit is measured by means of the Pearson cross-correlation coefficient between structure-derived (termed simulated density maps) and experimentally determined cryo-EM density maps. This procedure assigns a cross correlation value to each fragment, which is finally used for re-ranking of the top-100 list (Fig. 1c, ‘fitting fragment’). For generation of the simulated density maps for each suitable fragment (Fig. 1b, ‘map preprocessing’) the ‘copy from pdb’ functionality implemented in SPIDER was used [27]. The simulated density maps were subsequently filtered to the resolution of the experimental cryo-EM density map using a Butterworth low pass filter. Since procession time of cryo-EM density maps scales at least cubicly with image size, a minimal box enclosing the density of the queried segment is extracted from the cryo-EM density map (Additional file 1: Formula S1).

In the final preprocessing step, densities occupied by other parts of the structure are deleted from the minimal box (Fig. 1b). For that purpose, the part of the structure located within the minimal box is converted into a simulated density map with its intensity level adjusted to the value of the experimental map by a standard normalization (setting the average of the map to 0 and the standard deviation to 1). With a simple arithmetic operation, the simulated density map is subtracted from the minimal box reducing the cryo-EM density to the density of the missing fragment. Besides reducing procession time, this step limits false positive predictions by preventing placement of fragments into already occupied densities.

### Validation data set

For evaluation of FragFit, a test data set of cryo-EM density maps and structure coordinates of eight different macromolecular complexes selected from the EMDB [1] was composed. This data set (Table 1) includes proteins with different functions such as the ribosome, the proteasome and ion channels with resolutions ranging from 3.1 to 12 Å [7, 8, 28–33]. Using a sliding window of 5 to 35 amino acids length, a total of 20.000 different segments were assigned for evaluation. As for previous evaluations of fragment based approaches, fragments with sequence identities of more than 90% (for details see [12, 14]) to the queried segments were excluded from LIP prior calculations. This cut-off excludes identical structures, while keeping the conformational space as large as possible, thus mimicking a real life situation, where the best fitting fragment has to be selected from millions of candidates. Further, to assess the quality of FragSearch and FragFit (see Fig. 1) for prediction of different types of structural elements, helices, β-sheets and loops were assigned by means of the DSSP algorithm [34]. Finally, to estimate the impact of resolution on FragFit prediction quality, simulated density maps with resolutions ranging from 4 to 20 Å were used. Using simulated instead of experimentally determined cryo-EM density maps excludes bias by inhomogeneous resolutions or map fragmentation. Simulated electron density maps were calculated for the structure of the β2 adrenergic receptor–Gs protein complex (PDB-entry code: 3SN6) [35] using the ‘pdb_sim’ functionality of the NMFF program package [36]. As above, fragments with sequence identities of more than 90% were excluded from LIP prior calculations (for details see [12–14]).

**Table 1:** Structures and cryo-EM density maps used for evaluation of FragFit

EMDB-entry code	PDB-entry codes	Biological system	Resolution in Å	Citation
1721	3J59,3J5A	70S ribosome	12.0	(Bock et al., [28])
1798	2XSY,2XTG	70S ribosome	7.8	(Ratje et al., [31])
2490	4CE4	Mitochondrial large ribosomal subunit	4.9	(Greber et al., [29])
2566	3J6B	Mitochondrial large ribosomal subunit	3.2	(Amunts et al., [7])
5256	3IZX	cytoplasmic polyhedrosis virus	3.1	(Yu et al., [32])
2325	3ZPZ	GroEL/ES	8.9	(Chen et al., [33])
5776	3J5Q	TRPV1	3.8	(Cao et al., [8])
1733	3C91	20S proteasome	6.8	(Rabl et al., [30])

### Validation measures

The root mean square deviation (backbone-RMSD) was used as primary measure of structural similarity between an experimentally determined protein segment and its predicted conformation after superposition of the corresponding termini and stem atoms (Formula 1). Since only the backbone atoms but not the side chains are predicted, solely the coordinates of backbone atoms were used for evaluation. The difference of RMSD values of FragSearch and FragFit (ΔRMSD) was used to evaluate the gain in prediction quality, when cryo-EM density maps were used as restraints.

$$ \mathit{\mathsf{RMSD}}=\sqrt{{\frac{\mathsf{1}}{\mathit{\mathsf{N}}}}^{\ast}\sum \limits_{\mathit{\mathsf{i}}=\mathsf{1}}^{\mathit{\mathsf{N}}}{\left({\mathit{\mathsf{X}}}_{\mathit{\mathsf{i}}}-{\mathit{\mathsf{Y}}}_{\mathit{\mathsf{i}}}\right)}^{\mathsf{2}}} $$

Formula 1. Calculation of root mean square deviation (RMSD).

*N* is the number of atoms, *Xi* and *Yi* are the coordinates of the backbone atoms from both structures after superposition of the corresponding termini and stem atoms.

To provide a measure of similarity independent from the number of compared atoms, that is, of fragment length [37], the template modeling score (TM-score) was employed to assess the ‘topological similarity’ of two proteins (Formula 2a) [38]. The Method is described in detail in ref. [38]. Shortly summarized, the TM-score employs the length (‘*L’*) of the target protein and the number of aligned residues in both protein segments (‘Lali’) (see Formula 2a). The distance between each pair of aligned residues is *di*, while *d0* is a scaling value to normalize this match difference. The expression ‘*max’* denotes the maximum value after optimization of superposition. A simplified variation of the TM-score was used here (Formula 2b), since in our approach segments of identical length (‘Lali’ = *‘L’*) were used and no optimization of superposition of fragments was performed; only the stem residues were aligned. In principle, the value of the TM-score ranges from 0 to 1 with values of the TM-score > 0.5 denoting high topological similarity.

a $$ \mathit{\mathsf{TM}}-\mathit{\mathsf{score}}=\frac{\mathsf{1}}{\mathit{\mathsf{L}}}{\left[{\sum}_{\mathit{\mathsf{i}}=\mathsf{1}}^{{\mathit{\mathsf{L}}}_{\mathit{\mathsf{ali}}}}\frac{\mathsf{1}}{\mathsf{1}+\left({\mathit{\mathsf{d}}}_{\mathit{\mathsf{i}}}^{\mathsf{2}}/{\mathit{\mathsf{d}}}_{\mathsf{0}}^{\mathsf{2}}\right)}\right]}_{\mathit{\mathsf{max}}} $$

b $$ \mathit{\mathsf{TM}}-\mathit{\mathsf{score}}=\frac{\mathsf{1}}{\mathit{\mathsf{L}}}\left[{\sum}_{\mathit{\mathsf{i}}=\mathsf{1}}^{\mathit{\mathsf{L}}}\frac{\mathsf{1}}{\mathsf{1}+\left({\mathit{\mathsf{d}}}_{\mathit{\mathsf{i}}}^{\mathsf{2}}/{\mathit{\mathsf{d}}}_{\mathsf{0}}^{\mathsf{2}}\right)}\right] $$

Formula 2. a) General calculation of the TM-score, b) simplified Version used here.

## Results

To test the applicability of our fragment based approach for modeling of loops, helices or β-sheets into cryo-EM density maps, we evaluated the gain in prediction quality of classical fragment modeling when cryo-EM densities are employed as experimental restraints. For the initial step of fragment-based prediction (FragSearch) we employed the hierarchical search algorithm implemented in SL2 and the fragment database LIP [12–14]. In a second step we used the cross-correlation between simulated and experimentally determined density maps for re-scoring. The test data set includes functionally and evolutionary distinct proteins, whose structures were elucidated at resolutions between 3.1 Å and 12 Å by cryo-EM. We find a significant improvement of prediction quality depending on length and secondary structure of a missing segment as well as on the quality (resolution, fragmentation, noise) of cryo-EM density maps.

### Modeling accuracy of segments into cryo-EM density maps

**Fig. 2:**
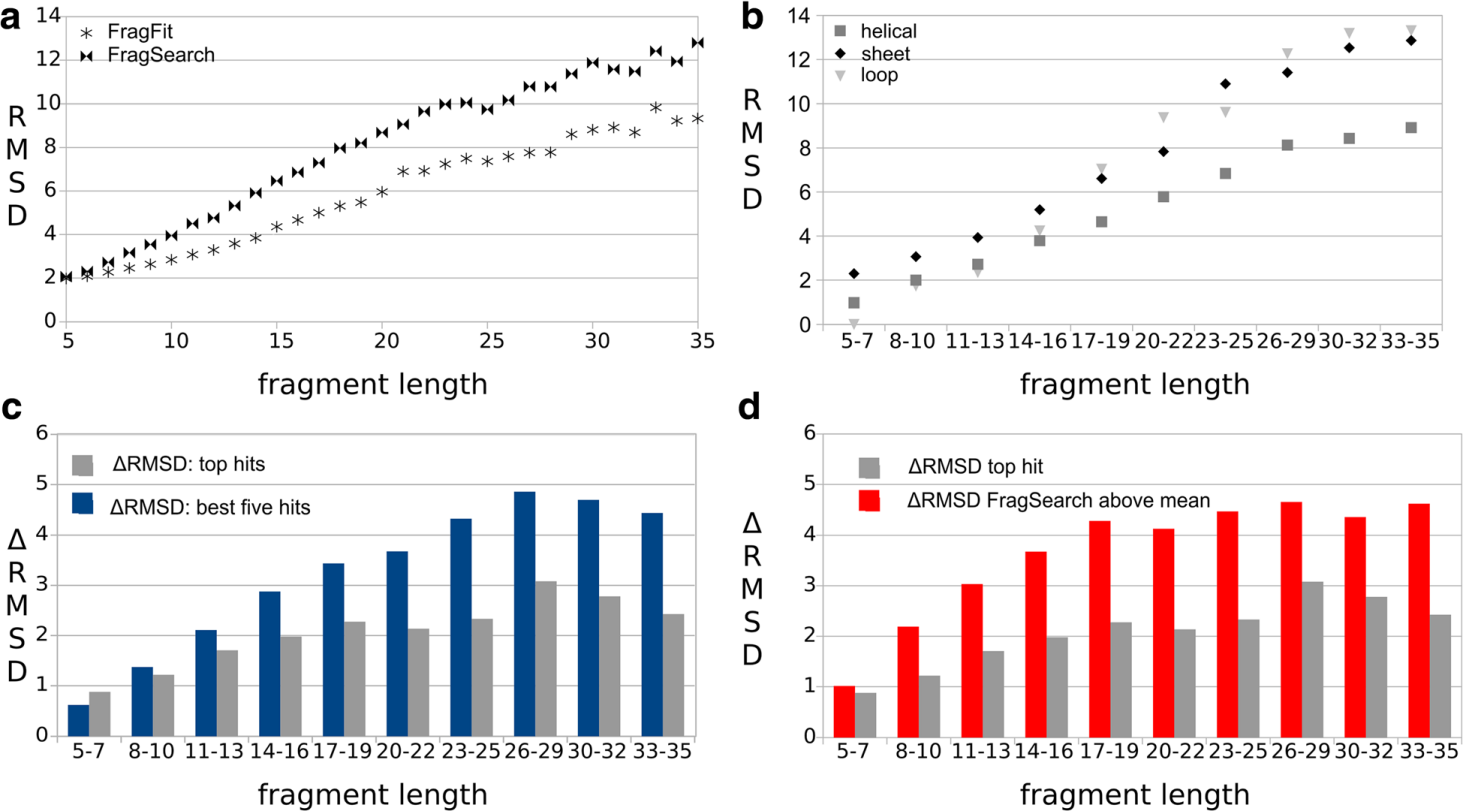
RMSD-based FragFit benchmarks. **a** Absolute backbone RMSD values of predicted fragment (top-hit) and original segment by FragSearch (double triangle) or FragFit (black star). **b** Comparison of absolute backbone RMSD values of predicted fragment (top-hit) and original segment by FragFit for the different structural elements helices (grey square), β-sheets (black rhombus) or loops (gray triangle). **c** Comparison of ΔRMSD (=RMSD FragSearch – RMSD FragFit) of top hit (gray bar) and top five hits (blue bars). **d** Comparison of ΔRMSD of top hits (gray bars) and only those top-hits were the RMSD of FragSearch is above the mean-value

The top-100 list of fragments is obtained by FragSearch, which uses the criteria sequence similarity and geometrical fit of stem atoms (see Fig. 1a). This top-100 list is re-scored by FragFit, which uses a cryo-EM density map as additional restraint. That step significantly improves prediction quality for all fragments longer than five residues (paired t-test with *P* ≤ 0.05). The absolute RMSD-values range from 1.9 Å for fragments with five residues length to 9.6 Å for fragments with 35 residues length (Fig. 2a). Modeling, therefore, improves on average by 1–2 Å (ΔRMSD) for fragments of 8–16 residues length and 2–3 Å (ΔRMSD) for longer fragments when cryo-EM density maps are employed (Fig. 2c, grey bars).

### Prediction quality depends on the secondary structure type

Prediction quality depends on the secondary structure type of the modeled segment. Helices, which become visible even at medium resolution cryo-EM density maps [5, 6], are found here as the secondary structure elements with highest predictability (Fig. 2b). When compared to other structural elements, the absolute RMSD value of helices is lower. This difference is more articulate for longer fragments. Loops, which here also include structural irregularities such as Pi-buldges or 3–10 helices, are predicted with similar accuracy as helices up to 16 residues length, before prediction quality drops down to the level of the β-sheets, which are generally most difficult to predict. The improvement of prediction of β-sheets and loops with FragFit is similar or even more pronounced as for helices up to a length of 25 residues but clearly drops for longer segments (Additional file 1: Figure S3).

### Prediction quality can be further enhanced when the top-five hits are taken into consideration

When not only the top hit but the top-five hits of FragFit (and FragSearch) are considered for evaluation, the performance is further improved by an additional average drop of the backbone-RMSD of about 1 Å (Fig. 2c). This benefit is again particularly pronounced for longer fragments. For fragments of e.g. 17 amino acids length, the mean backbone RMSD to the original segment drops from 7.2 Å (top hit FragSearch) and 5.0 Å (top hit FragFit) to 3.9 Å (top-five hit FragFit). For fragments of 27 amino acids length, the corresponding values are 10.1 Å (top hit FragSearch), 7.2 Å (top hit FragFit) and 5.6 Å (top-five hit FragFit). When additional hits are taken into account (e.g. top-ten hits FragFit), no further improvement is obtained (Additional file 1: Figure S4) suggesting that the best solution is regularly found within the top five results list.

Furthermore, a significant gain in prediction quality is observed with FragFit when only those FragSearch top-hits were considered with an RMSD above the mean RMSD (indicated as double triangles in Fig. 2a). In those cases, the gain in prediction quality measured by the drop of the backbone-RMSD is about 2 Å larger as the gain when all FragSearch top-hits were considered (Fig. 2d). This result suggests that the gain in prediction quality largely stems from down ranking of fragments with non native conformations.

### FragFit selects for the right fold

The backbone-RMSD was used as a measure of structural similarity. Specifically, we measured the average distance between the backbone atoms of a selected fragment and the original protein segment after superposition of the corresponding termini and stem atoms (see Methods). Using this measure, all atoms are taken into account with equal weight. For high RMSD-values typically observed with longer fragments it, however, remains unclear whether this value stems from similar structures with local deviations (such as a kink) or completely different structures/folds.

To provide a second quality assessment for evaluation of longer fragments, we employed the TM-score, which is designed as a measure of similarity in structure or fold. This measure is also considered to be rather independent of protein length [39]. A TM-score > 0.5 indicates a similar structure or fold. Our analysis of the TM-score provides evidence that fragments with appropriate structure are regularly identified by FragFit, especially for fragments up to 25 residues length. For fragments longer than 12 amino acids we find that the TM-score between original and predicted fragment (top hit FragFit) is higher than 0.5 in 81% of predictions. In 82–93% of predictions of fragments of 12–25 residue length a similar structure is found. The number of fragments with a score higher then 0.5-score drops to values of 69–76% for fragments of 26–35 residue length (Additional file 1: Figure S5). According to the TM-score analysis, the conformation of fragments up to 25 residues length can be predicted with high accuracy.

### Influence of resolution on fragment prediction quality

**Fig. 3:**
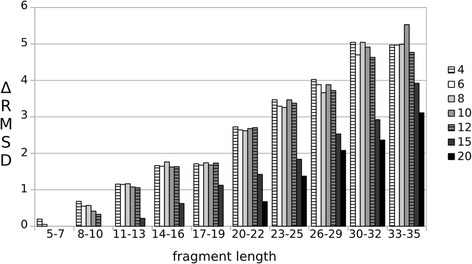
ΔRMSD between FragSearch and FragFit for simulated cryo-EM maps of different resolutions. The gain of FragFit over FragSearch is constant for resolutions ranging from 4 to 12 Å for fragments of at least 12 residues length. Only a minor improvement of prediction quality is obtained with resolutions of 15 Å or 20 Å for segments of at least 11 or 20 residues length, respectively

Assessment of the influence of resolution on fragment prediction quality is complicated, because of local variations in structure resolution and fragmentation of cryo-EM density maps. To estimate the influence of resolution on prediction quality, we generated simulated density maps from the X-ray structure of the β2 adrenergic receptor-Gs protein complex (PDB accession code: 3SN6) with resolutions ranging from 4 to 20 Å (Fig. 3). This membrane protein complex contains 35% helices, 19.2% sheets and 45.8% unassigned regions, such as loops or kinks, thus representing the complete relevant spectrum of protein secondary structures evaluated here. The advantage of using simulated instead of experimentally determined cryo-EM density maps is that factors which would influence this analysis such as noise or fragmentation are excluded. Of note, the PDB entry 3SN6 and all fragments with a sequence identity of more than 90% have been excluded from the fragment database.

As with experimentally determined cryo-EM density-maps the gain in prediction quality (ΔRMSD) increases with fragment length (Fig. 3). Only for the highest resolution maps of 4–6 Å, a minor improvement of prediction quality is also seen for the short fragments of 5–7 residues length. A constant increase in prediction quality up to ΔRMSD = 5 Å is seen for simulated density maps of 4–12 Å resolutions for fragments of 8–35 residues length. For the low resolution maps of 15 and 20 Å, a minor gain in prediction quality is only observed for segments of at least 11 or 20 residues length, respectively. The higher gain in prediction quality of simulated compared to experimentally determined density maps shows how noise and fragmentation of experimentally determined cryo-EM density-maps complicates modeling. In summary, FragFit performs very well over a wide range of resolutions but best for high- and medium resolution maps.

## Discussion

Using a representative data set of protein structures resolved by cryo-EM, we provide evidence that fragment based approaches can be applied to model protein segments into cryo-EM density maps at high accuracy. Our results are complementary to previous approaches using cryo-EM density maps for rigid [40–42] or flexible fitting [43–45] of existing structures, or for de novo modeling of complete protein structures into high resolution cryo-EM density maps [46]. One outstanding feature is that FragFit, which uses the same hierarchical strategy to find suitable fragments as SL2 [12–14], provides results within one or few minutes even for long fragments (depending on box size and running environment). This renders FragFit applicable for web-based applications providing easy access for structural biologists.

FragFit can be used to model or remodel parts of proteins. It has been proven to guide modeling of poorly resolved flexible loops in ribosome bound initiation factor-2, which cryo-EM density map was resolved at 3.7 Å resolution. Initial models generated by FragFit were verified or optimized by real-space refinement in Phenix 1.10 [10]. Moreover, FragFit can be readily integrated into modeling approaches, where conformational changes of proteins only affect a substructure of the protein or a single domain, while the general fold remains unchanged [11]. In these cases, flexible fitting of the complete structure or complex is not required. Instead, the structure can be dissembled into its different domains which are rigidly fitted [40]. FragFit can then be used to reconnect these domains or to re-model the hinge regions. Since the fragments are taken from PDB structures which have undergone several steps of quality control, the fragments do not necessarily have to be refined, only the side chain rotamers may have to be edited. Moreover, automatically refinement tools as Rosetta [47], or a short energy minimization might be used to further improve the completed structure with regards to the newly ligated backbone stem atoms, which may suffer from small structural distortions due to geometrical inconsistencies.

**Fig. 4:**
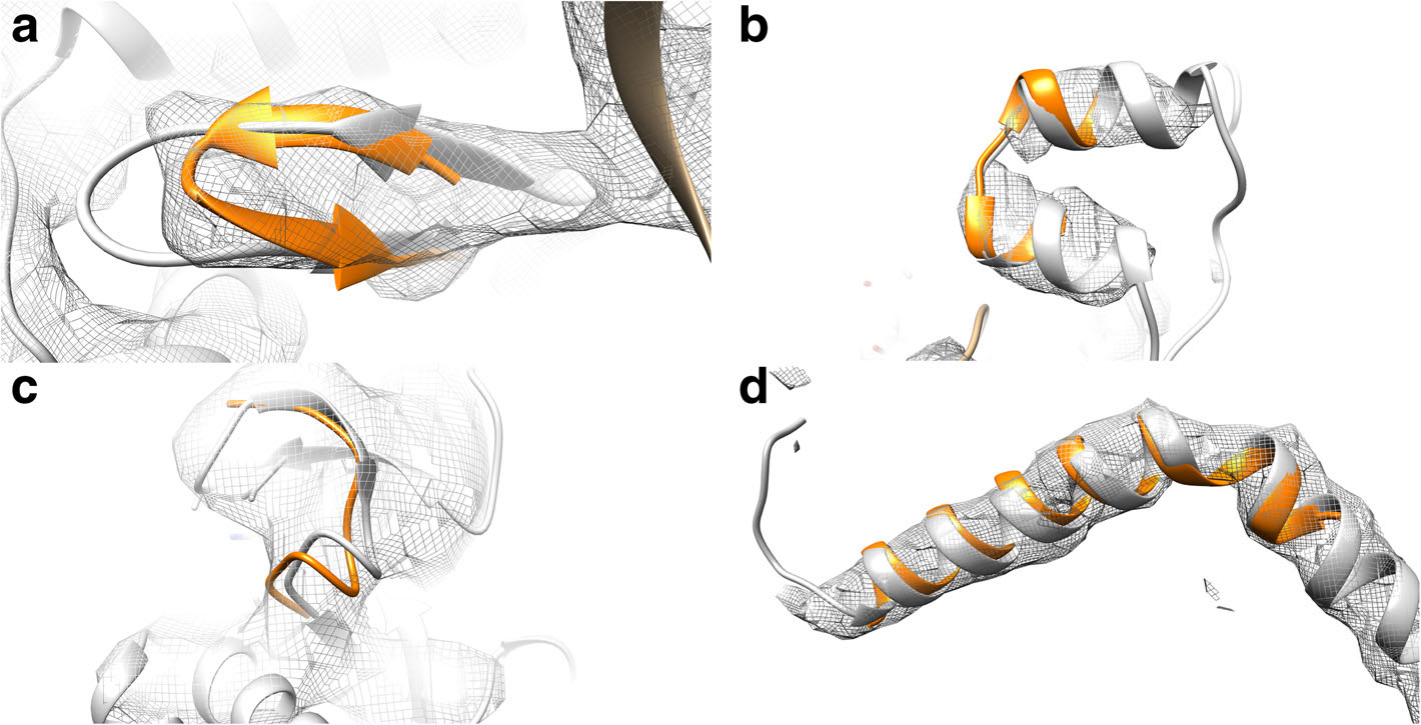
FragFit examples. **a** A 12 residue long β-sheet from Ribosomal protein L28 (PDB 2XTG, template PDB 3FZL with 25% sequence identity). **b** TRPV1 ankyrin repeat region (PDB 3J5Q, template PDB 3EU9, sequence identity 23%). **c** Loop in GroEL connecting two β-sheets (PDB 3ZPZ, template PDB 3RTK with 26% sequence identity).**d** Long helix in TRPV1 (PDB 3J5Q,template PDB 3R2P with 19% sequence identity). Originally fitted structures are colored gray, fragments found by FragFit are colored orange

The accuracy of FragFit depends on the type of secondary structure and of the quality (resolution, fragmentation, noise) of the map. The high reliability of prediction of helices can be explained by the characteristic sequence composition and geometry of α-helices, that are often well defined and clearly visible in cryo-EM density maps. By contrast, β-sheets and long loops, that are stabilized by more complex tertiary or quaternary structure interactions involving residues distant in primary structure, are much more difficult to model and to identify even in medium resolution maps [48]. Despite this fact, analysis of the TM-score suggests that FragFit is also capable of modeling β-sheets and complex loop structures, particularly when a homologous template structure is available (Fig. 4b, c).

The gain in prediction quality is higher in those cases, where FragSearch was unable to select the best fragment (Fig. 2d, ΔRMSD FragSearch fails). Our analysis, therefore, reveals that false positives are cleaned out from the top- (Fig. 2d) and the top-five results list (Fig. 2c), when cryo-EM density maps are used as restraints. An additional gain in prediction quality is obtained, when the top-five results list is taken into account. Visualization of the top-five fragments is therefore expected to aid selection of the best fitting fragment, particularly in case of fragmented maps or maps with unassigned but not relevant densities. Fragmentation might in several cases thus impact modeling quality more than overall resolution. If noise or fragmentation is absent, resolution of 12 Å would theoretically be sufficient to guide the modeling process (Fig. 3). In this case, even low resolution maps support modeling of segments longer than 20 residues, suggesting that if the rough shape of the queried segment is defined by the map the native conformation could be selected from the ensemble of conformations suggested by FragSearch. Finally, fragmentation might in part also refer to the presence of an ensemble of different conformations rather than one well defined state. Loops of proteins are often highly flexible and split up into various substates with sub-micro second lifetimes [49]. In these cases FragFit might be useful to contour the possible ensemble of different conformations present in flexible protein regions.

## Conclusion

In summary, FragFit has proven to be a valuable tool for the modeling of protein segments into cryo-EM density map. Particularly for longer segments, cryo-EM density maps add additional restraint that improve classical fragment based modeling. The low requirements in computing power recommend implementation of FragFit for instant visualization in web-applications (runtime approximately within a few minutes, depending on the running environment, fragment length and box size). Visual control allows interactive selection of the most appropriate fragment, which we consider as a necessary step to select for the most appropriate conformation, specifically when artifacts or map fragmentations complicate fully automatic modeling. The database LIP and the programs FragSearch and FragFit are accessible on request.

## Additional file

Additional file 1: Supplementary Figures S1-S6 and Formula 1. (DOCX 1069 kb)

## Abbreviations

cryo-EM: Electron cryo-microscopy
EMDB: Electron Microscopy Data Bank
LIP: Fragment database ‘Loops in Proteins’
PDB: Protein Data Bank
RMSD: Root-mean-square deviation
ΔRMSD: RMSD FragSearch – RMSD FragFit

## References

[1] Lawson CL, Baker ML, Best C, Bi C, Dougherty M, Feng P (2011). EMDataBank.Org: unified data resource for CryoEM. Nucleic Acids Res.

[2] Mahdavi S, Salehzadeh-Yazdi A, Mohades A, Masoudi-Nejad A (2013). Computational structure analysis of biomacromolecule complexes by interface geometry. Comput Biol Chem.

[3] Villa E, Lasker K (2014). Finding the right fit: chiseling structures out of cryo-electron microscopy maps. Curr Opin Struct Biol.

[4] Baker ML, Baker MR, Hryc CF, Ju T, Chiu W (2012). Gorgon and pathwalking: macromolecular modeling tools for subnanometer resolution density maps. Biopolymers.

[5] Baker ML, Ju T, Chiu W (2007). Identification of secondary structure elements in intermediate-resolution density maps. Structure.

[6] Si D, Ji S, Nasr KA, He J (2012). A machine learning approach for the identification of protein secondary structure elements from electron cryo-microscopy density maps. Biopolymers.

[7] Amunts A, Brown A, Bai XC, Llacer JL, Hussain T, Emsley P (2014). Structure of the yeast mitochondrial large ribosomal subunit. Sci (80- ).

[8] Cao E, Liao M, Cheng Y, Julius D (2013). TRPV1 structures in distinct conformations reveal activation mechanisms. Nature.

[9] Brown A, Long F, Nicholls RA, Toots J, Emsley P, Murshudov G (2015). Tools for macromolecular model building and refinement into electron cryo-microscopy reconstructions. Acta Crystallogr D Biol Crystallogr.

[10] Sprink T, Ramrath DJ, Yamamoto H, Yamamoto K, Loerke J, Ismer J (2016). Structures of ribosome-bound initiation factor 2 reveal the mechanism of subunit association. Sci Adv.

[11] Chapman BK, Davulcu O, Skalicky JJ, Brüschweiler RP, Chapman MS (2015). Parsimony in protein conformational change. Structure.

[12] Ismer J, Rose AS, Tiemann JK, Goede A, Preissner R, Hildebrand PW (2016). SL2: an interactive webtool for modeling of missing segments in proteins. Nucleic Acids Res.

[13] Michalsky E, Goede A, Preissner R (2003). Loops in proteins (LIP)--a comprehensive loop database for homology modelling. Protein Eng.

[14] Hildebrand PW, Goede A, Bauer R a, Gruening B, Ismer J, Michalsky E (2009). SuperLooper - a prediction server for the modeling of loops in globular and membrane proteins. Nucleic Acids Res.

[15] Rose AS, Elgeti M, Zachariae U, Grubmüller H, Hofmann KP, Scheerer P (2014). Position of transmembrane helix 6 determines receptor G protein coupling specificity. J Am Chem Soc.

[16] Choi Y, Deane CM (2010). FREAD revisited: accurate loop structure prediction using a database search algorithm. Proteins.

[17] Fiser A, Do RK, Sali A (2000). Modeling of loops in protein structures. Protein Sci.

[18] Kelm S, Vangone A, Choi Y, Ebejer JP, Shi J, Deane CM (2014). Fragment-based modeling of membrane protein loops: successes, failures, and prospects for the future. Proteins.

[19] Rose PW, Bi C, Bluhm WF, Christie CH, Dimitropoulos D, Dutta S (2013). The RCSB protein data Bank: new resources for research and education. Nucleic Acids Res.

[20] Mandell DJ, Coutsias EA, Kortemme T (2009). Sub-angstrom accuracy in protein loop reconstruction by robotics-inspired conformational sampling. Nat Methods.

[21] Tang K, Zhang J, Liang J (2014). Fast protein loop sampling and structure prediction using distance-guided sequential chain-growth Monte Carlo method. PLoS Comput Biol.

[22] Liu T, Horst JA, Samudrala R (2009). A novel method for predicting and using distance constraints of high accuracy for refining protein structure prediction. Proteins.

[23] Boomsma W, Mardia KV, Taylor CC, Ferkinghoff-Borg J, Krogh A, Hamelryck T (2008). A generative, probabilistic model of local protein structure. Proc Natl Acad Sci U S A.

[24] Zhao S, Zhu K, Li J, Friesner RA (2011). Progress in super long loop prediction. Proteins.

[25] Du P, Andrec M, Levy RM (2003). Have we seen all structures corresponding to short protein fragments in the protein data Bank? An update. Protein Eng.

[26] Fernandez-Fuentes N, Fiser A (2006). Saturating representation of loop conformational fragments in structure databanks. BMC Struct Biol.

[27] Frank J, Radermacher M, Penczek P, Zhu J, Li Y, Ladjadj M (1996). SPIDER and WEB: processing and visualization of images in 3D electron microscopy and related fields. J Struct Biol.

[28] Bock LV, Blau C, Schroder GF, Davydov II, Fischer N, Stark H (2013). Energy barriers and driving forces in tRNA translocation through the ribosome. Nat Struct Mol Biol.

[29] Greber BJ, Boehringer D, Leitner A, Bieri P, Voigts-Hoffmann F, Erzberger JP (2014). Architecture of the large subunit of the mammalian mitochondrial ribosome. Nature.

[30] Rabl J, Smith DM, Yu Y, Chang SC, Goldberg AL, Cheng Y (2008). Mechanism of gate opening in the 20S proteasome by the proteasomal ATPases. Mol Cell.

[31] Ratje AH, Loerke J, Mikolajka A, Brunner M, Hildebrand PW, Starosta AL (2010). Head swivel on the ribosome facilitates translocation by means of intra-subunit tRNA hybrid sites. Nature.

[32] Yu X, Ge P, Jiang J, Atanasov I, Zhou ZH (2011). Atomic model of CPV reveals the mechanism used by this single-shelled virus to economically carry out functions conserved in multishelled reoviruses. Structure.

[33] Zhuang T, Chen Q, Cho M-K, Vishnivetskiy S a, Iverson TM, Gurevich VV (2013). Involvement of distinct arrestin-1 elements in binding to different functional forms of rhodopsin. Proc Natl Acad Sci U S A.

[34] Kabsch W, Sander C (1983). Dictionary of protein secondary structure: pattern recognition of hydrogen-bonded and geometrical features. Biopolymers.

[35] Rasmussen SGF, Choi H-J, Fung JJ, Pardon E, Casarosa P, Chae PS (2011). Structure of a nanobody-stabilized active state of the β(2) adrenoceptor. Nature.

[36] Tama F, Miyashita O, Brooks CL (2004). Flexible multi-scale fitting of atomic structures into low-resolution electron density maps with elastic network normal mode analysis. J Mol Biol.

[37] Carugo O, Pongor S (2001). A normalized root-mean-square distance for comparing protein three-dimensional structures. Protein Sci.

[38] Zhang Y, Skolnick J (2004). Scoring function for automated assessment of protein structure template quality. Proteins.

[39] Xu J, Zhang Y (2010). How significant is a protein structure similarity with TM-score = 0.5?. Bioinformatics.

[40] Kawabata T (2008). Multiple subunit fitting into a low-resolution density map of a macromolecular complex using a gaussian mixture model. Biophys J.

[41] Tjioe E, Lasker K, Webb B, Wolfson HJ, Sali A (2011). MultiFit: a web server for fitting multiple protein structures into their electron microscopy density map. Nucleic Acids Res.

[42] Woetzel N, Lindert S, Stewart PL, Meiler J (2011). BCL::EM-fit: rigid body fitting of atomic structures into density maps using geometric hashing and real space refinement. J Struct Biol.

[43] Trabuco LG, Villa E, Schreiner E, Harrison CB, Schulten K (2009). Molecular dynamics flexible fitting: a practical guide to combine cryo-electron microscopy and X-ray crystallography. Methods.

[44] Wang Z, Schroder GF (2012). Real-space refinement with DireX: from global fitting to side-chain improvements. Biopolymers.

[45] Whitford PC, Ahmed A, Yu Y, Hennelly SP, Tama F, Spahn CM (2011). Excited states of ribosome translocation revealed through integrative molecular modeling. Proc Natl Acad Sci U S A.

[46] Wang RY, Kudryashev M, Li X, Egelman EH, Basler M, Cheng Y (2015). De novo protein structure determination from near-atomic-resolution cryo-EM maps. Nat Methods.

[47] Wang RY-R, Song Y, Barad BA, Cheng Y, Fraser JS, DiMaio F (2016). Automated structure refinement of macromolecular assemblies from cryo-EM maps using Rosetta. Elife.

[48] Si D, He J (2014). Tracing beta strands using StrandTwister from cryo-EM density maps at medium resolutions. Structure.

[49] Elgeti M, Rose AS, Bartl FJ, Hildebrand PW, Hofmann KP, Heck M (2013). Precision vs flexibility in GPCR signaling. J Am Chem Soc.

