# Supplementary material for: A fragment based method for modeling of protein segments into cryo-EM density maps
Source: BMC Bioinformatics. 2017 Nov 13;18:475. doi: 10.1186/s12859-017-1904-5 (PMC5683378; doi:10.1186/s12859-017-1904-5)
Supplement: Additional file 1: — Supplementary Figures S1-S6 and Formula 1. (DOCX 1069 kb) [file 12859_2017_1904_MOESM1_ESM.docx]

Supplemental material

A fragment based method for modeling of protein segments into cryo-EM density maps

# by Jochen Ismer^1^, Alexander S. Rose^1,2^, Johanna K. S. Tiemann^1,3^ and Peter W. Hildebrand^1,4,*^

^1^Institute of Medical Physics and Biophysics, University Medicine Berlin, Charitéplatz 1, 10117 Berlin, Germany

^2^present address: Alexander S. Rose, San Diego Supercomputer Center, University of California, San Diego, CA 92093-0743, USA

^3^present address: Johanna K. S. Tiemann, Institute of Medical Physics and Biophysics, University Leipzig, Härtelstraße 16-18, 04107 Leipzig, Germany

^4^second address: Peter W. Hildebrand, Institute of Medical Physics and Biophysics, University Leipzig, Härtelstraße 16-18, 04107 Leipzig, Germany

*Correspondence: peter.hildebrand@charite.de, peter.hildebrand@medizin.uni-leipzig.de


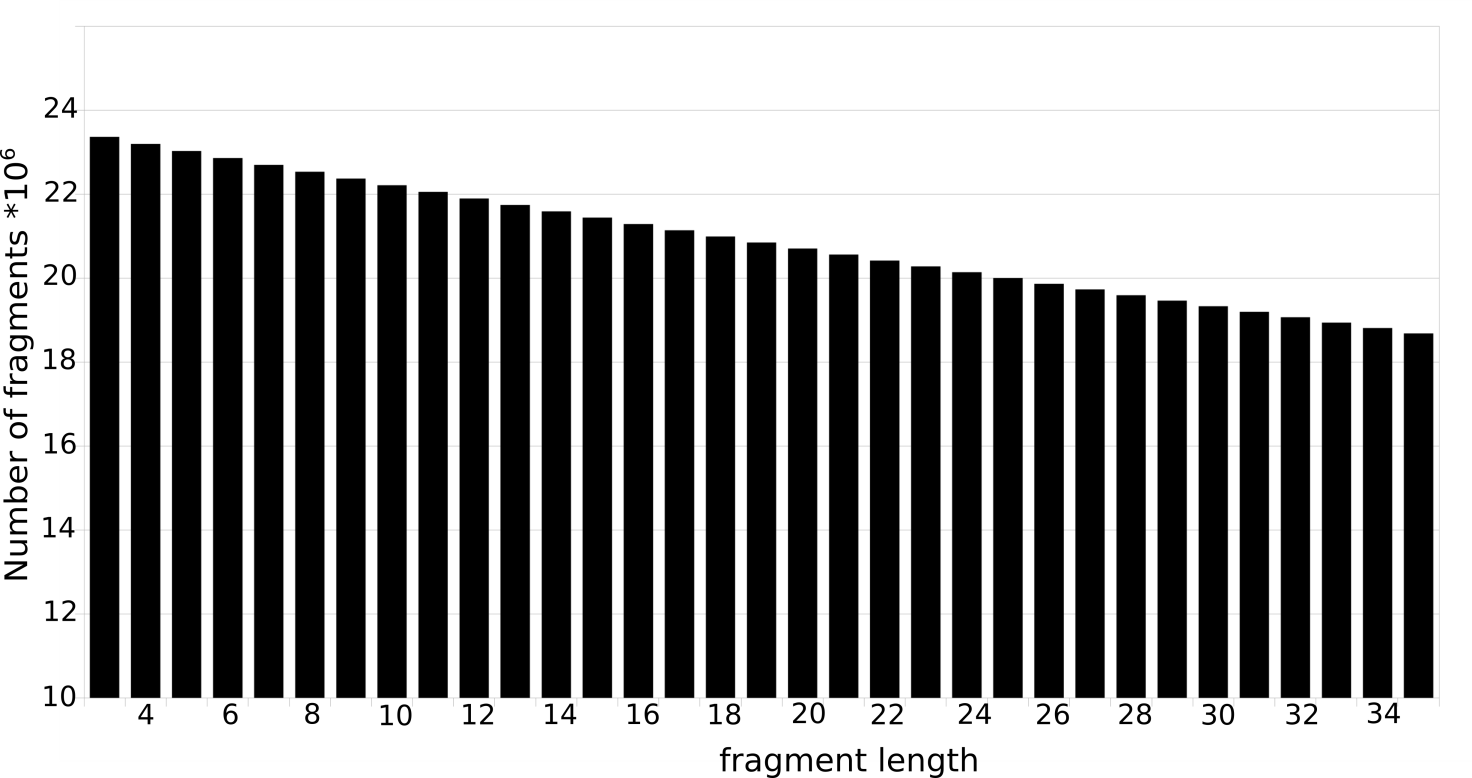


**Fig. S1. Length distribution of fragments stored in LIP in 2013 :** From the about 100.000 structures deposited in the PDB (state June 2013), 9*10^8^ overlapping fragments with 3-35 residue length were extracted. The number of fragments decreases with fragment length.


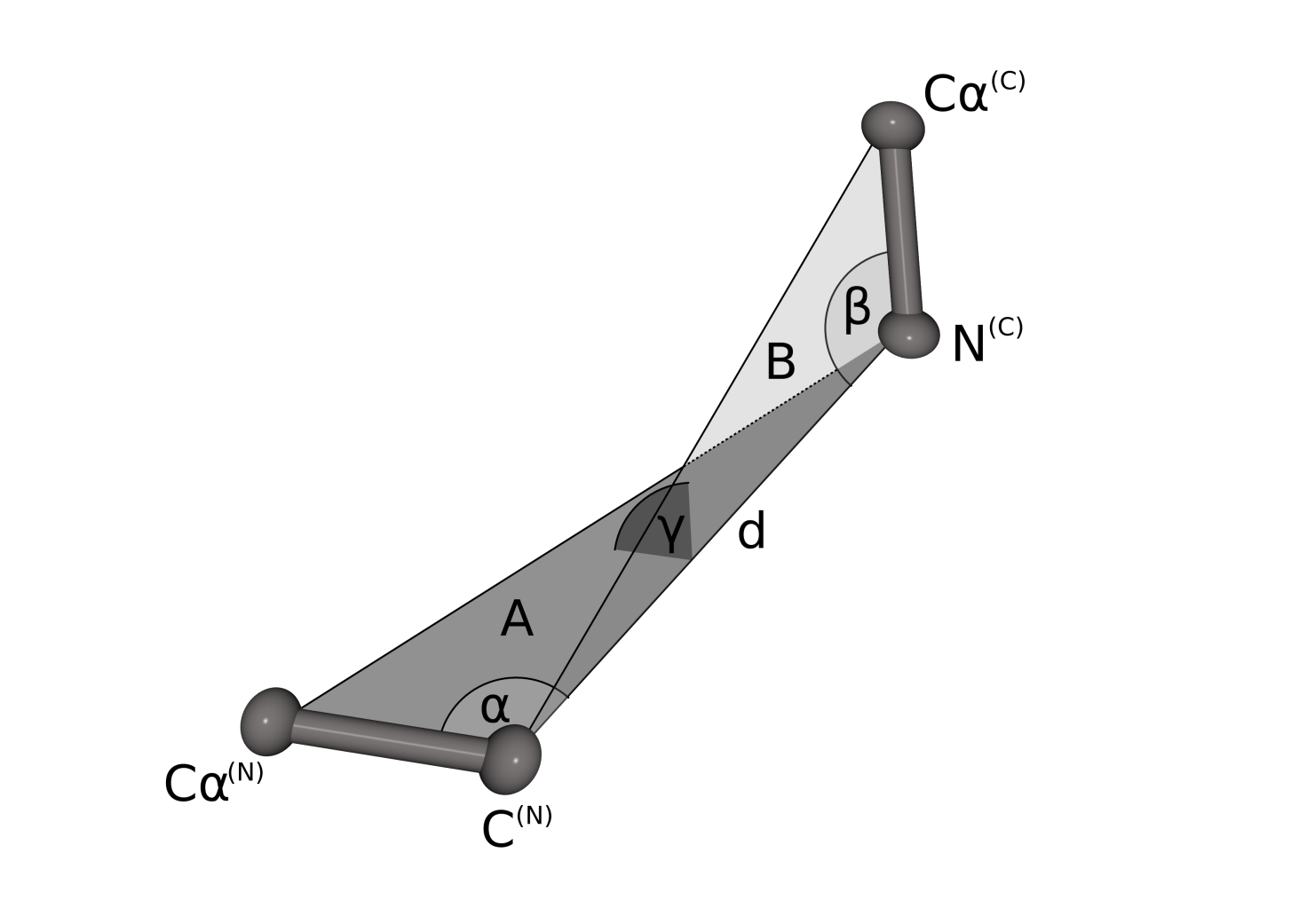


**Fig. S2. Schematic representation of the geometrical fingerprint:** The geometrical fingerprint is characterized by the distance d between the N-terminal C- and the C-terminal N atom and the following three angles: α defined by the line between Cα^(N)^ and C^(N)^ and d, β defined by the line between N^(C)^ and Cα^(C)^ and d, γ the angle between the two planes A (defined by Cα^(N)^, C^(N)^ and N^(C)^) and B (Cα^(C)^, C^(N)^ and N^(C)^).


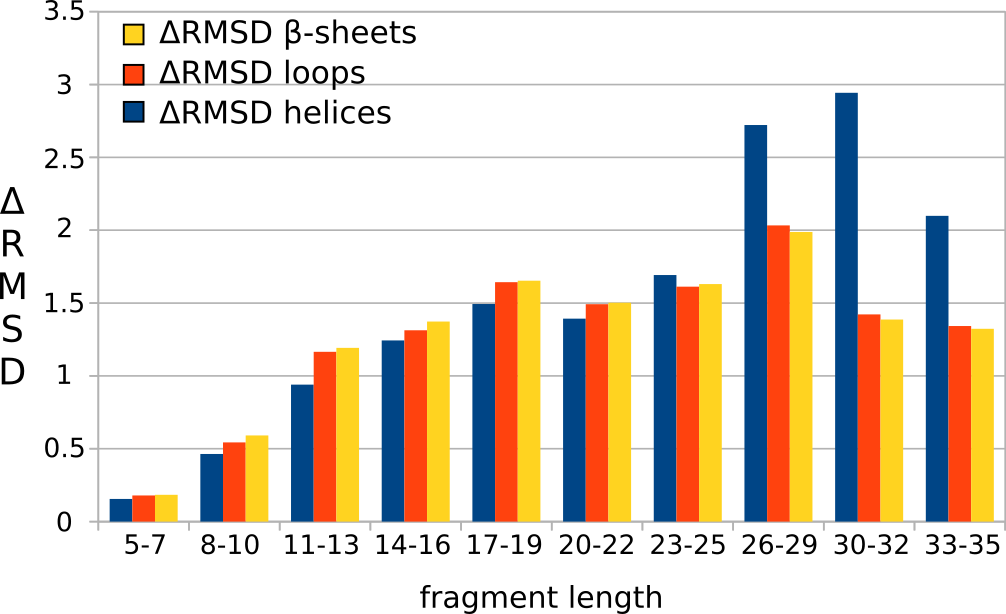


**Fig. S3.Improvement of prediction quality by secondary structure**: Comparison of ΔRMSD (=RMSD FragFit – RMSD FragSearch) of top hit sorted by the secondary structure type. β-sheets (yellow bars), loops (orange bars) and helices (blue bars).


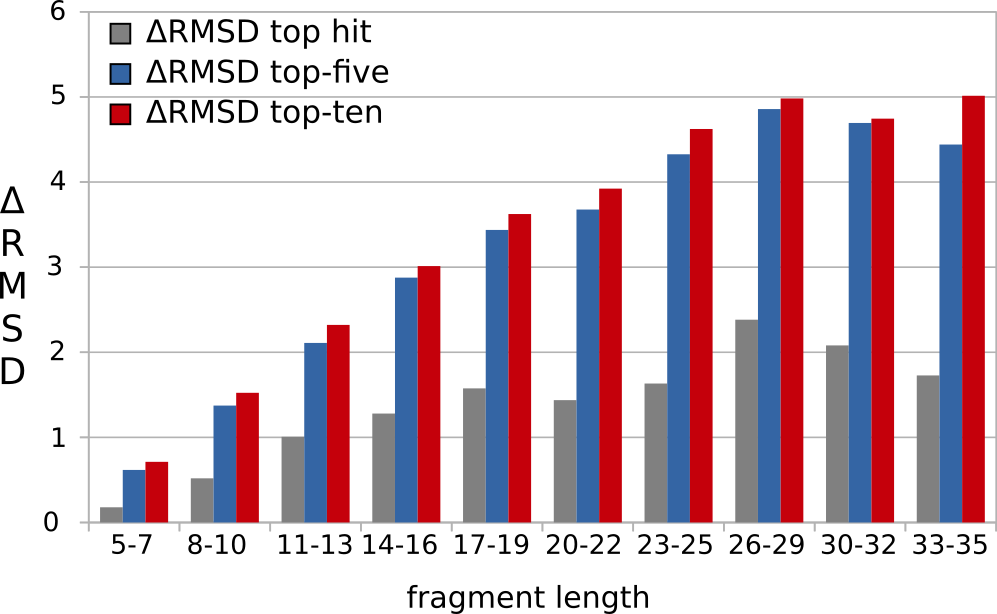


**Fig S4:** Comparison of ΔRMSD (=RMSD FragFit – RMSD FragSearch) of top hit (grey bar), top five hits (blue bars) and top ten hits (red bars). While the gain in prediction quality using the top five list is significant, the additional gain in using the top ten list is negligible.


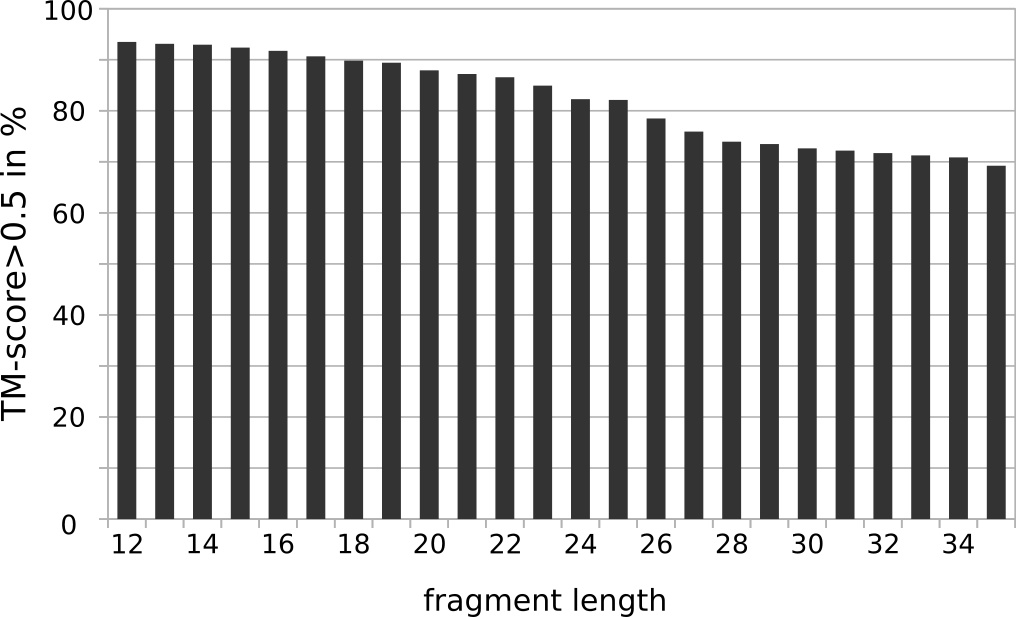


**Fig S5. Proportion (in %) of FragFit top hits with TM-Score > 0.5 sorted by fragment length:** A score > 0.5 denotes high topological similarity between predicted and experimentally determined conformation.


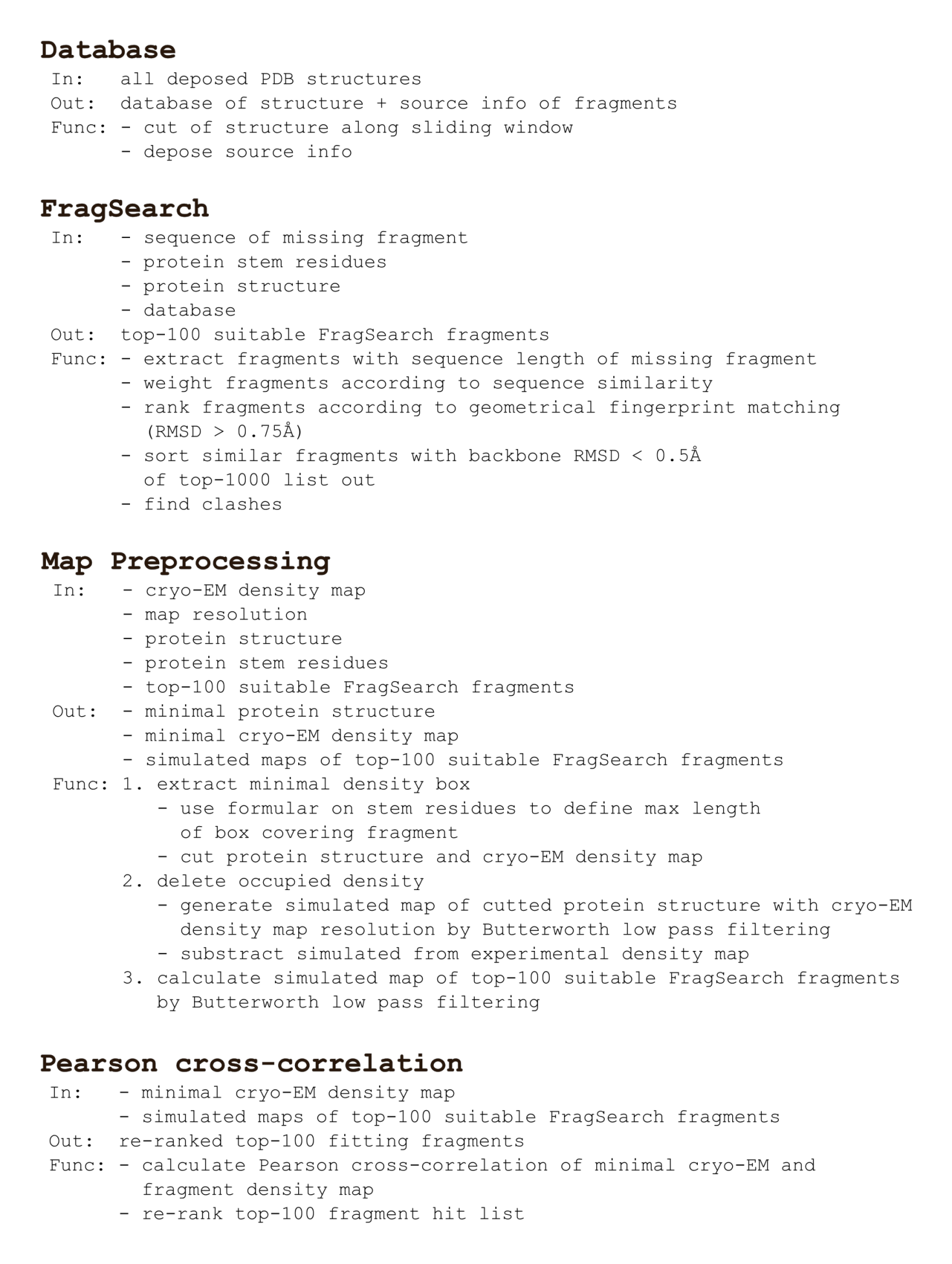


**Fig S6. Pseudocode of the FragFit workflow.**

Since procession time of cryo-EM density maps scales at least cubicly with image size, a minimal box enclosing the density of the queried segment is extracted from the cryo-EM density map. For that purpose, a minimal box centered between the two stem atoms is defined as a cuboid with a lateral length X.

$$X=\left( \frac{a}{2}*w \right)-d$$

Suppl. Formula 1: Formula defining the lateral length of the minimal box used to reduce the density to the queried segment. 'a' is the length of the segment, 'w' is a factor that converts the extension of each residue into its pixel size (pixel spacing) and 'd' the distance between the two stem-residues of the gap. As a result the expansion of the minimal box is provided in pixel size.
